# Probing Out‐Of‐Plane Charge Transport in Organic Semiconductors Using Conductive Atomic Force Microscopy

**DOI:** 10.1002/adma.202418694

**Published:** 2024-12-26

**Authors:** Mindaugas Gicevičius, Haoxin Gong, Nicholas Turetta, William Wood, Martina Volpi, Yves Geerts, Paolo Samorì, Henning Sirringhaus

**Affiliations:** ^1^ Cavendish Laboratory University of Cambridge JJ Thomson Avenue Cambridge CB3 0HE UK; ^2^ Université de Strasbourg CNRS ISIS UMR 7006 8 allée Gaspard Monge Strasbourg F‐67000 France; ^3^ Laboratoire de Chimie des Polymères Université Libre de Bruxelles (ULB) Boulevard du Triomphe, CP 206/01 Bruxelles 1050 Belgium; ^4^ International Solvay Institutes of Physics and Chemistry Université Libre de Bruxelles (ULB) CP 206/01, Boulevard du Triomphe, CP231 Bruxelles 1050 Belgium

**Keywords:** conductive atomic force microscopy, contact resistance, high mobility, organic semiconductors

## Abstract

High contact resistance remains the primary obstacle that hinders further advancements of organic semiconductors (OSCs) in electronic circuits. While significant effort has been directed toward lowering the energy barrier at OSC/metal contact interfaces, approaches toward reducing another major contributor to overall contact resistance – the bulk resistance – have been limited to minimizing the thickness of OSC films. However, the out‐of‐plane conductivity of OSCs, a critical aspect of bulk resistance, has largely remained unaddressed. In this study, multi‐layered 2D crystalline, solution‐processed films of the high‐mobility molecular semiconductor 2,9‐dioctylnaphtho[2,3‐b] naphtha[2′,3′:4,5]thieno[2,3‐d]thiophene (C8‐DNTT‐C8) are investigated using conductive‐probe atomic force microscopy (C‐AFM) to evaluate out‐of‐plane charge transport. The findings reveal a linear increase in out‐of‐plane resistance with the number of molecular layers in the film, which is modeled using an equivalent circuit model with multiple tunneling barriers connected in series. Building upon these results, a vertical transfer length method (V‐TLM) is developed, allowing one to determine the out‐of‐plane resistivity of OSC and providing insights into charge transport properties at a single molecule length scale. The V‐TLM approach highlights the potential of C‐AFM for investigating out‐of‐plane charge transport in OSC thin films and holds promise for accelerating the screening of molecules for high‐performance electronic devices.

## Graphical abstract

Ultra‐thin, layered single crystal films of molecular semiconductor 2,9‐dioctylnaphtho[2,3‐b]naphtha[2′,3′:4,5]thieno[2,3‐d]thiophene (C8‐DNTT‐C8) are studied using conductive atomic force microscopy (C‐AFM), revealing changes in out‐of‐plane electrical current as a function of the number of molecular layers. A vertical transfer length method is devised, which enables to estimate the out‐of‐plane charge transport properties of an organic semiconductor at an unprecedented molecular length scale.

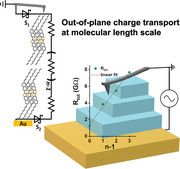

## 1 Introduction

Recent decades have been marked by substantial progress in the development of organic semiconductors (OSCs), with their in‐plane charge‐carrier mobilities – a commonly used figure of merit – nowadays routinely exceeding 10 cm^2^ V^−1^ s^−1^ in the best performing materials.^[^ ^1^, ^2^, ^3^ ^]^ However, despite promising applications, further advances in electronic circuits based on OSCs have been hampered by high contact resistances that limit the efficiency of charge injection and extraction through metallic contacts.^[^ ^4^, ^5^ ^]^

It has been shown that certain chemical modifications to fused aromatic systems, such as the introduction of alkyl chain substituents, can improve solution processability and enhance the in‐plane charge carrier mobility of molecular OSCs by suppressing large amplitude intermolecular vibrations, thus leading to some of the best performing molecular OSC systems to date.^[^ ^6^, ^7^ ^]^ However, while these modifications benefit in‐plane charge transport, the introduction of electrically insulating side chains may negatively affect performance in staggered architecture devices.^[^ ^8^ ^]^ Specifically, they increase contact resistance in such devices, where charge carriers have to travel through the bulk of the semiconducting layer before reaching the accumulation layer at the interface with the gate dielectric layer.^[^ ^9^ ^]^ This has been demonstrated in alkylated (dinaphtho[2,3‐*b*:2′,3′‐*f*]thieno[3,2‐*b*]thiophene) DNTT derivatives, where an increase in side‐chain length was found to improve the in‐plane mobility, but had a pronounced negative effect on the contact resistance in high‐performance monolayer organic field‐effect transistor (OFET) devices.^[^ ^10^ ^]^

The contact resistance, *R*_C_, in staggered electronic devices consists of two main contributions: the interface (*R*_I_) and bulk (*R*_bulk_) resistances (i.e., *R*_C_ *= R*_I_ *+ R*_bulk_). *R*_I_ arises from the Schottky barrier at the metal/OSC interface, while *R*_bulk_ originates from the bulk resistance of the OSC layer. Conventional approaches for lowering contact resistance generally focus on the OSC/metal interface engineering, aiming to minimize the Schottky barrier. This is usually achieved either by modifying the work function of the electrode (for instance, with self‐assembled monolayers)^[^ ^11^, ^12^, ^13^ ^]^ and/or by contact doping for improved contact/OSC energy‐level alignment.^[^ ^14^ ^]^ However, these approaches address only one part of the problem, as another major contributor to high contact resistances is the bulk resistivity of the OSC. Considering that bulk resistance scales with the thickness of the OSC layer, it is not surprising that the best‐performing OFET devices reported recently had ultra‐thin active channels consisting of only 1–2 molecular layers of material, in which the role of bulk resistance is minimized.^[^ ^5^, ^10^, ^15^, ^16^ ^]^

While efforts to maximize in‐plane mobilities have been mainly focused on the optimization of molecular design and the synthesis of novel OSC derivatives, the problem of bulk resistance has been largely unaddressed from a molecular engineering perspective. This gap can mostly be attributed to a lack of advanced techniques and models for studying charge transport in the out‐of‐plane direction. In this study, we investigate 2D crystalline films of 2,9‐dioctylnaphtho[2,3‐b]naphtha[2′,3′:4,5]thieno[2,3‐d]thiophene (C8‐DNTT‐C8) and demonstrate how conductive‐probe atomic force microscopy (C‐AFM) can be used to evaluate the out‐of‐plane charge transport properties of multi‐layered molecular semiconductor films. C‐AFM has already been demonstrated to be a powerful tool for studying molecular junctions,^[^ ^17^ ^]^ grain boundaries,^[^ ^18^ ^]^ as well as an in‐plane charge transport within a single OSC grain.^[^ ^19^ ^]^ Here, we used C‐AFM to map the local changes in electrical current through multi‐layered structures of OSC and developed a vertical transfer length method (V‐TLM) to determine the out‐of‐plane resistivity of C8‐DNTT‐C8. We report a novel microscopic approach based on mapping local variations in current in multi‐layered OSC films, thus gaining insights into the charge transport properties at an unprecedented, single molecule length scale.

## 2 Results and Discussion

**Figure 1:**
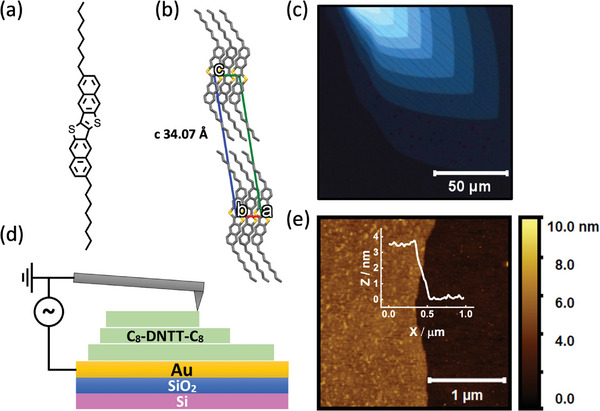
a) Molecular structure and b) crystal structure of C8‐DNTT‐C8. c) Polarized optical microscopy image of a multi‐layered C8‐DNTT‐C8 film prepared by the solution shearing method. d) Schematics of C‐AFM measurement. e) AFM topography scan of C8‐DNTT‐C8 film step edge and its corresponding height profile (inset).

In this study, we investigated the local electrical properties of solution‐processed organic thin films of the molecular semiconductor C8‐DNTT‐C8, whose chemical structure is presented in **Figure** **1**a. C8‐DNTT‐C8 is a di‐alkylated derivative of the molecular semiconductor dinaphtho[2,3‐b:2’,3’‐f]thieno[3,2‐b]thiophene (DNTT) bearing octyl chains on its terminal ends. Compared to its unsubstituted predecessor, C8‐DNTT‐C8 shows improved solubility in organic solvents and enhanced self‐assembly properties that enable the deposition of highly crystalline films with large‐area crystal domains, using facile solution‐based techniques. In its crystal form, C8‐DNTT‐C8 adopts a lamellar structure with the DNTT cores forming high‐mobility, herringbone‐stacked molecular planes parallel to the substrate, separated by electrically insulating octyl side chains (Figure 1b).^[^ ^7^ ^]^

We employed a meniscus‐guided solution‐shearing method to deposit highly crystalline C8‐DNTT‐C8 films on Si/SiO_2_ wafers coated with uniform, thermally evaporated Au film and investigated their morphological and electrical properties using polarized optical microscopy (POM) and atomic force microscopy (AFM). The POM image in Figure 1c shows large‐area single‐crystal domains with a multi‐layered structure in which every layer has the same crystal orientation. The self‐assembly of C8‐DNTT‐C8 molecules is highly sensitive to the surface roughness of the underlying substrate. For this reason, Si/SiO_2_ wafers, known for their atomically flat surfaces, were used to deposit Cr/Au layers with a low surface roughness, facilitating the formation of highly ordered C8‐DNTT‐C8 films (Figure S1, Supporting Information). Surface contamination or defects of Cr/Au layers (Figure S1b, Supporting Information) may have led to the formation of pinholes in the first few molecular layers of our C8‐DNTT‐C8 film, as observed in the POM image in Figure 1c and the later AFM scan images. Likewise, the microcracks in the C8‐DNTT‐C8 films likely occur due to a mismatch of thermal expansion coefficients between the substrate and the OSC.^[^ ^20^ ^]^

To investigate local out‐of‐plane charge transport properties in C8‐DNTT‐C8, we carried out a comprehensive analysis by recording the topography and electrical current maps using conductive atomic‐force microscopy (C‐AFM). A schematic representation of C‐AFM measurements for multi‐layered C8‐DNTT‐C8 films is illustrated in Figure 1d. An electrically conducting AFM probe is brought into contact with the crystalline film, and a voltage bias is applied between it and the underlying Au film. The resulting flow of current through the probe is recorded using a current‐to‐voltage preamplifier.

From height‐map data, an average step height of 3.4 nm was observed between neighboring crystal terraces, as shown in Figure 1e. This value corresponds well to the unit cell *c*‐axis length of C8‐DNTT‐C8 in the bulk phase (*c* = 34.07 Å, CCDC: 1845516) and confirms that the molecules in the film are aligned with their long axes perpendicular to the substrate. The thickness of a single‐crystal film can therefore be expressed as *n* × 3.4 nm, where *n* is the number of C8‐DNTT‐C8 molecular layers.

**Figure 2:**
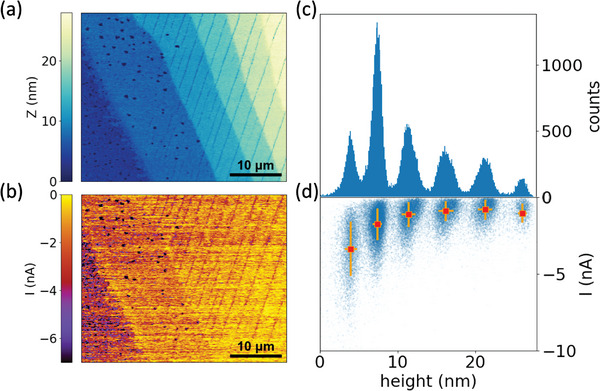
C‐AFM topography (a) and electrical current (b) maps of a multi‐layered single crystal C8‐DNTT‐C8 film. The C‐AFM measurements were performed at −1.0 V sample bias and 15 nN mechanical load. c) Height histogram of C8‐DNTT‐C8 film AFM topography scan. d) Electrical current versus height plot of multi‐layered C8‐DNTT‐C8 film. The error bars indicate standard deviations. The striations in the topography and current maps are due to thermal micro‐cracks in the C8‐DNTT‐C8 crystalline film.

**Figure** **2**a,b displays C‐AFM height and electrical current maps, respectively, of a region within a C8‐DNTT‐C8 sample containing multiple molecular layers. These maps show a clear correlation between their respective features. For instance, there is a noticeable difference in the magnitude of electrical current between crystal terraces of differing heights, while the currents remain relatively uniform within the same layer. These results suggest that each OSC layer in the film acts as a resistor in series for currents flowing in the out‐of‐plane direction. Figure 2c presents a histogram of the C‐AFM height‐map values, showing a clear clustering of heights, which indicates the presence of well‐defined molecular layers in the C8‐DNTT‐C8 film. The height and current values from each point of the C‐AFM scan were then plotted against each other in Figure 2d. Here, a clear clustering is observed, with the centroid of each cluster demonstrating that the average value of the out‐of‐plane current decreases as the number of layers *n* increases.

**Figure 3:**
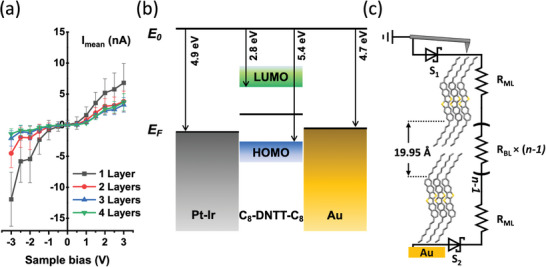
a) Current‐voltage measurements of C8‐DNTT‐C8 films of different thickness obtained via C‐AFM. b) Band diagram of the AFM tip/C8‐DNTT‐C8/Au structure. *E*_0_ marks the vacuum level, *E*_F_ marks the Fermi level, and LUMO denotes the lowest unoccupied molecular orbital energy level of C8‐DNTT‐C8. c) Equivalent electronic‐circuit model of the AFM tip/C8‐DNTT‐C8/Au structure for the multi‐layered crystalline C8‐DNTT‐C8 film.

We varied the bias applied to the sample to record current‐voltage characteristics of Pt‐Ir/C8‐DNTT‐C8/Au junctions. Due to noise inherent to the measurement, single‐point *I‐V* curves were insufficient for providing representative data. Therefore, averaging over multiple positions was necessary. To achieve this, we recorded C‐AFM maps under different applied voltage biases and plotted the mean current value for each terrace height against the applied bias, as shown in **Figure** **3**a. These *I‐V* curves exhibit pronounced non‐linearities, indicating a rectifying behavior, which is a feature of some metal‐semiconductor‐metal (MSM) structures and is typically attributed to the presence of Schottky barriers. At the Pt‐Ir/OSC (i.e., the probe tip) and OSC/Au interfaces these barriers arise due to the energy‐level mismatch between the highest occupied molecular orbital (HOMO) level of C8‐DNTT‐C8 and the work function of the metal electrodes (Figure 3b). Similar to DNTT and its other alkylated derivatives, C8‐DNTT‐C8 has a deep HOMO level, making it relatively stable under ambient conditions, which enabled us to perform C‐AFM measurements in air.^[^ ^21^ ^]^ As with other OSC materials under similar conditions, trace levels of oxygen doping can be expected.^[^ ^22^ ^]^ However, we believe that any effect of trace oxygen doping on out‐of‐plane charge transport in our C‐AFM measurements is likely to be typical of device operating conditions in the field. The influence of trace oxygen doping will be the subject of future investigations.

Given the deep HOMO level of C8‐DNTT‐C8 which aligns relatively closely with the work function of the metal contacts (Pt‐Ir, Au) used in the junction, efficient hole injection is expected. In combination with a likelihood of a light p‐type doping by oxygen at trace levels, we thus consider holes to be the dominant charge carriers in the C‐AFM measurement. Consequently, when a positive sample bias is applied, holes are injected through the Au‐coated substrate and extracted at the conducting AFM probe, while the reverse holds true at negative sample bias values. Measurable current flow was only observed when the applied bias exceeded a certain threshold voltage, which was determined by linear extrapolation of the above‐threshold *I‐V* characteristics. Under positive bias the positive threshold voltage was $V_{\mathrm{th}}^{\mathrm{pos}}=0.65\;V$, while under negative bias, when the hole injecting Schottky diode at the tip/OSC interface is reverse‐biased, a higher threshold voltage of $V_{\mathrm{th}}^{\mathrm{neg}}=-0.83\;V$ was observed (Figure 3a). A more detailed discussion on the effects of sample bias polarity is presented later in a later section.

Given that the film is composed of conjugated, semiconducting DNTT units separated by unconjugated, insulating alkyl (C8‐) side chains, we consider charge transport in the out‐of‐plane direction to be predominantly governed by a sequence of electronic tunneling events through the interposing alkyl chains, which act as barriers between the high‐mobility planes formed by the DNTT units. This mechanism is consistent with previous studies on molecular OSCs featuring non‐conjugated side chains.^[^ ^23^, ^24^ ^]^ As tunneling probability decreases exponentially with increasing barrier width, the barrier imposed by paired alkyl chains between two DNTT layers is considerably greater than that of the unpaired alkyl chains at the top and bottom of the film. Consequently, the bulk resistance of the OSC film, *R*_bulk_, is dominated by the resistance of alkyl side‐chain bilayers, whose number can be expressed as (*n –* 1).^[^ ^23^, ^24^ ^]^ In a well‐ordered crystalline C8‐DNTT‐C8 film, a bilayer of octyl side chains creates an insulating layer with a width of 19.95 Å, as shown in Figure 3b. Previous studies have reported that tunneling through the mono‐ and bi‐layers of alkyl chains primarily takes place through bond, rather than through layer, with an average barrier height of *Φ*_B_ **=** 1.4 eV.^[^ ^25^ ^]^

Based on these considerations, we devised an equivalent electronic circuit model to describe the C‐AFM measurements of multi‐layered films of C8‐DNTT‐C8, as illustrated in Figure 3c. The total electrical resistance, *R*_tot_, through such a circuit can then be expressed using the following equation:

(1) $$R_{\mathrm{tot}}=R_{S1}\left(V\right)+R_{S2}\left(V\right)+2R_{\mathrm{ML}}+\left(n-1\right)R_{\mathrm{BL}}$$

where *R*_S1,S2_ *(V)* are the bias‐dependent resistances attributed to the Schottky barriers at the two OSC/metal interfaces, *R*_ML_ is the resistance of each unpaired alkyl‐chain layer, and *R*_BL_ is the electrical resistance of each paired alkyl‐chain layer.

The extraction of the equivalent circuit parameters by fitting the *I‐V* curve cannot be performed analytically and requires a numerical solution. These calculations are often complicated by the effects at the metal/OSC interfaces, such as the presence of interfacial states and Fermi‐level pinning.^[^ ^26^ ^]^ Consequently, we found that the most suitable regions of the crystalline film for studying the out‐of‐plane charge transport are those characterized by a large step density, i.e. having multiple terraced layers within the AFM scan area (e.g. 40 µm × 40 µm). This combination of sampling area dimensions and topographic features yields a sufficiently large dataset of electrical current values as a function of the film thickness, represented by the number of molecular layers, *n*.

**Figure 4:**
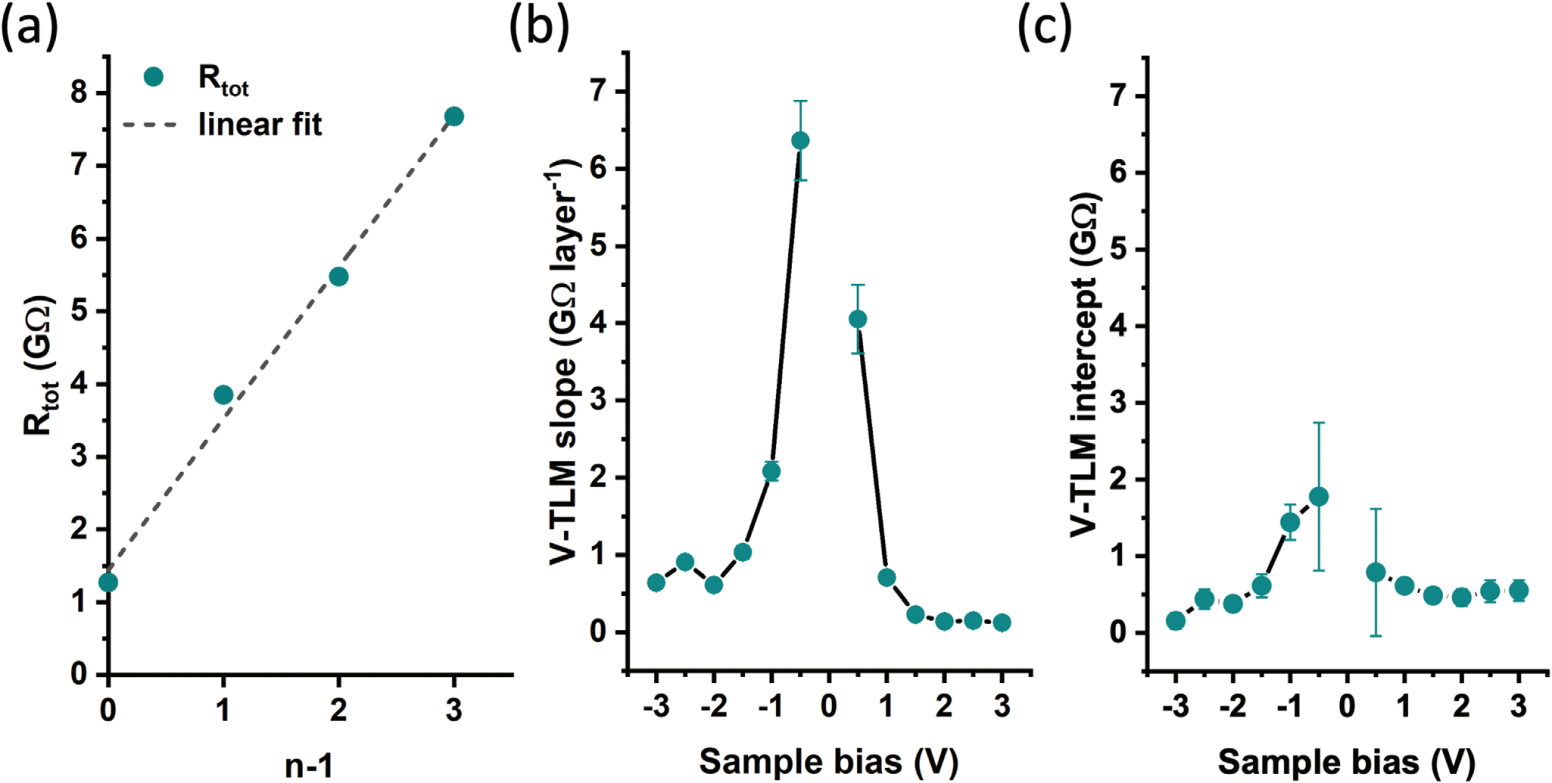
a) Vertical transfer length method (V‐TLM) plot of C‐AFM total resistance (*R*_tot_) versus the number of alkyl bilayers in the film (*n* – 1) at −1.0 V sample bias and ≈15 nN mechanical load. b) V‐TLM plot slope values representing the increase in *R*_tot_ when the thickness of C8‐DNTT‐C8 is increased by a single molecular layer, plotted as a function of voltage bias applied to the sample. c) V‐TLM intercept values representing the resistance of the metal/OSC interfaces and the resistance of the first OSC monolayer as a function of *V*_sample_.

Using the C‐AFM measurement data from Figure 2d, we plotted the mean total resistance (*R*_tot_ = *V*/*I*_mean_) for each individual crystal layer as a function of (*n* – 1) (**Figure** **4**a). We observed a linear increase in *R*_tot_ (R^2^ > 0.99) with each additional molecular layer (increasing *n*). This apparent linear dependence between the out‐of‐plane resistance and thickness (*n*) suggests that the charge transport in our experiments is not in a space‐charge‐limited current (SCLC) regime, which has previously been used to extract out‐of‐plane charge carrier mobility in C‐AFM measurements.^[^ ^27^, ^28^ ^]^ SCLC transport in planar device geometries is described by the Mott‐Gurney law which predicts a power‐law relation between resistance and thickness of *R*∝*L* ^3^. For measurements in C‐AFM geometry this was revised to *R*∝*L* ^1.4 ± 0.1^ by Reid et al. in 2008.^[^ ^27^ ^]^ However, those measurements were performed on films of greater thickness than those used in our experiments. In contrast, the linear increase of *R*_tot_ vs. (*n* – 1) observed in this work is characteristic of a set of identical Ohmic resistors connected in series. This finding is in good agreement with previously reported studies on solution‐processed, multilayered OSC films, where an analogous linear voltage drop with an increasing number of crystalline layers was observed over an extended film thickness range of 1 < *n* ≤ 15.^[^ ^23^, ^24^ ^]^ C‐AFM measurements of thicker C8‐DNTT‐C8 films with *n* > 4 are introduced in a later section.

While our work focuses on out‐of‐plane transport, measurements of total resistance as a function of channel length (*L*) are commonly used in the in‐plane direction in transistor devices to extract the values of contact resistance (*R*_C_) and sheet resistance (*R*_sh_).^[^ ^4^, ^5^, ^29^ ^]^ Equivalently, in our measurements, the variation in the number of layers (*n*) corresponds to a variation in channel length in the vertical (out‐of‐plane) direction. Therefore, Figure 4a can be interpreted as a “vertical transfer‐length method” (V‐TLM) plot, where the contribution of each additional molecular layer to the total resistance (*R*_tot_) is represented by the slope between neighboring data points. Since the change in *R*_tot_ in the V‐TLM plot (i.e. the slope) depends solely on *n*, this method allows us to estimate the intrinsic electrical properties of the OSC, independent of interfacial contact effects. Considering this, our proposed C‐AFM‐based V‐TLM approach offers a molecular‐scale view into the out‐of‐plane charge transport properties of molecular OSCs.

The y‐intercept of the linear V‐TLM plot represents the total C‐AFM resistance at *n* = 1, which corresponds to a combination of the probe/OSC and OSC/substrate interface resistances, along with the out‐of‐plane resistance of the first OSC monolayer. Thus, the y‐intercept value in the V‐TLM plot signifies the smallest achievable value of contact resistance when the OSC film thickness is reduced to a single monolayer.

The V‐TLM model is predicated on the assumptions that the probe/OSC and OSC/substrate interfaces remain stable during the C‐AFM scan, and that the stacked molecular layers are structurally and electrically identical. The validity of these assumptions is supported by relatively uniform electrical current maps within each C8‐DNTT‐C8 layer and the near‐perfect linearity of the V‐TLM plot shown in Figure 4a. Deviations from these assumptions such as AFM probe degradation or molecular layer inhomogeneity could introduce non‐linearity in the plot, affecting the accuracy of the out‐of‐plane charge transport properties analysis.

By applying different sample bias to the same region, its effect on the slope and intercept of the resulting V‐TLM plots was investigated, as shown in Figure 4b,c. The large slope values that can be seen at low voltages correspond to the sub‐threshold region of the *I‐V* characteristics (as depicted in Figure 3a), where *R*_tot_ is dominated by the resistance of the interfacial Schottky barriers. When the sample bias exceeds the threshold voltage, the V‐TLM slope values plateau at 160 ± 50 MΩ layer^−1^ for positive biases and 800 ± 180 MΩ layer^−1^ for negative sample voltage. This discrepancy between layer resistance values at different bias polarity is accompanied by the decrease in the goodness of linear fit (*R* ^2^) at higher positive sample bias values (*V*_sample_ > +1.5 V), and corresponds to different hole injection scenarios, which will be discussed in more detail in the following section.

Meanwhile, the y‐intercept values in Figure 4c, remain relatively similar at both positive (510 ± 40 MΩ) and negative (400 ± 160 MΩ) sample voltages, indicating that the measured interfacial resistance and the resistance of the first monolayer are less dependent on bias polarity than the bulk resistance.

**Figure 5:**
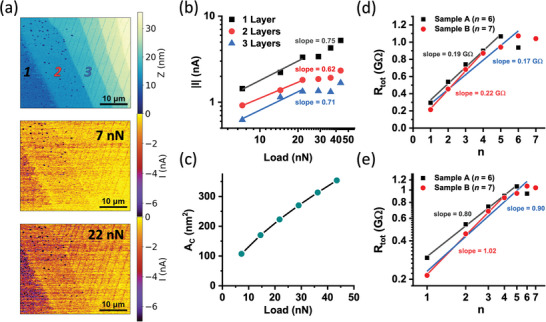
a) C‐AFM topography and electrical current maps of C8‐DNTT‐C8 film at different mechanical loads applied by the AFM probe (*V*_sample_ = −1.0 V). b) A log‐log plot depicting the mean current through the crystalline C8‐DNTT‐C8 layers of different thickness as a function of mechanical load applied by the AFM probe. c) The AFM probe/C8‐DNTT‐C8 junction area calculated using Hertz theory, plotted against the applied mechanical load. d) C‐AFM *R*_tot_ *vs. n* plot of C8‐DNTT‐C8 films with higher thickness of *n* = 6 (sample A), and *n =* 7 (sample B). e) Log‐log plot and linear fit of C‐AFM measurements of *R*_tot_ *vs. n* of C8‐DNTT‐C8 samples A and B (*V*_sample_ = −1.0 V, *P* ≈ 29 nN).

To calculate physically relevant resistivity values for the Pt‐Ir/C8‐DNTT‐C8/Au junction, the effective electrical contact area of the AFM probe/OSC junction needs to be estimated. The total measured current should scale with the probe/OSC junction area which, in turn, depends on the mechanical load applied by the probe (**Figure** **5**a). Figure 5b shows the mean current passing through C8‐DNTT‐C8 layers of different thickness during the C‐AFM scans recorded under different mechanical loads. Assuming that the increase in electrical current is solely due to an increased contact area, we used the Hertz contact model to estimate the AFM probe contact area.^[^ ^30^ ^]^ The Hertz theory of elastic contacts predicts that at low mechanical loads (*P*) the contact area (*A_C_*) between a spherical probe and a plane follows a power law: $A_{C}\propto P^{2/3}$. In our results, an average exponent value of 0.69 was extracted (by fitting a straight line to the log‐log plot) for all layers measured under small mechanical loads below 25 nN, indicating that the electrical contact area can indeed be estimated using the Hertz model.

For an applied mechanical load *P*, and probe radius *R*, the Hertz theory predicts that the radius *a* of elastic contact follows the relation:

(2) $$a^{3}=\frac{3PR}{4E^{*}}$$

where:

(3) $$\frac{1}{E^{*}}=\frac{\left(1-\vartheta_{1}^{2}\right)}{E_{1}}+\frac{\left(1-\vartheta_{2}^{2}\right)}{E_{2}}$$

and where *E*_1,2_ are the elastic moduli, and *ϑ*_1,2_ are the Poisson ratios of Pt‐Ir and C8‐DNTT‐C8, respectively. We used *E* = 200 GPa, and *ν* = 0.38 for the Pt‐Ir‐coated probe,^[^ ^31^ ^]^ and the nominal tip radius of 25 nm. For C8‐DNTT‐C8 we estimated the Poisson ratio to be *ν* = 0.33 and measured its Young's modulus as 0.61 ± 0.10 GPa by recording force‐distance curves (Figure S2, Supporting Information). The measured elastic modulus of C8‐DNTT‐C8 is lower than the reported elastic moduli of unsubstituted molecular organic semiconductors (≈15 GPa in Pentacene) but higher than the Young's modulus of alkanethiols of similar chain length (0.28 GPa in 1‐octanethiol).^[^ ^32^, ^33^, ^34^ ^]^ The C‐AFM contact area (*A*_C_) can then be calculated as a surface area of a spherical cap:

(4) $$A_{C}=\pi \left(a^{2}+\delta^{2}\right)$$

where *δ* is the elastic indentation depth, which becomes negligible at low mechanical loads. Using this approach, we estimated a contact area of 171 nm^2^ for the probe/OSC junction under a 15 nN mechanical load (Figure 5c), which at a molecular density of 4.25 molecules/nm^2^ corresponds to ≈730 molecules of C8‐DNTT‐C8 within the area of the junction.

Due to the noise floor of our C‐AFM setup, the measurable thickness range of C8‐DNTT‐C8 thin films was limited. At higher n values (*n* > 4), the measured resistance (*R*_tot_) starts to deviate from linearity due to the worsening signal‐to‐noise ratio. To overcome this limitation, and to demonstrate the linearity of *R*_tot_ over a wider *n* range, we increased the out‐of‐plane current by applying a greater mechanical force with the AFM probe (≈29 nN), thereby effectively increasing the probe/OSC contact area. This adjustment allowed for accurate C‐AFM measurements on two thicker films of C8‐DNTT‐C8: samples A and B with max(*n*) = 6 and 7 respectively. This allowed us to confirm the linear trend of *R*_tot_ *vs. n* over an extended thickness range, up to *n* = 6 (Figure 5d). The corresponding C‐AFM topography and current maps, along with the *I* versus *Z* plots for samples A and B are presented in Figure S3 (Supporting Information).

Moreover, to further demonstrate this linearity, we plotted *R*_tot_ against *n* for samples A and B on logarithmic scales, as shown in Figure 5e. Power laws, when plotted on logarithmic axes, appear as straight lines, with their gradients corresponding to the exponent values. Since the gradients are close to 1, this confirms the linearity of *R*_tot_ with respect to *n*.

**Figure 6:**
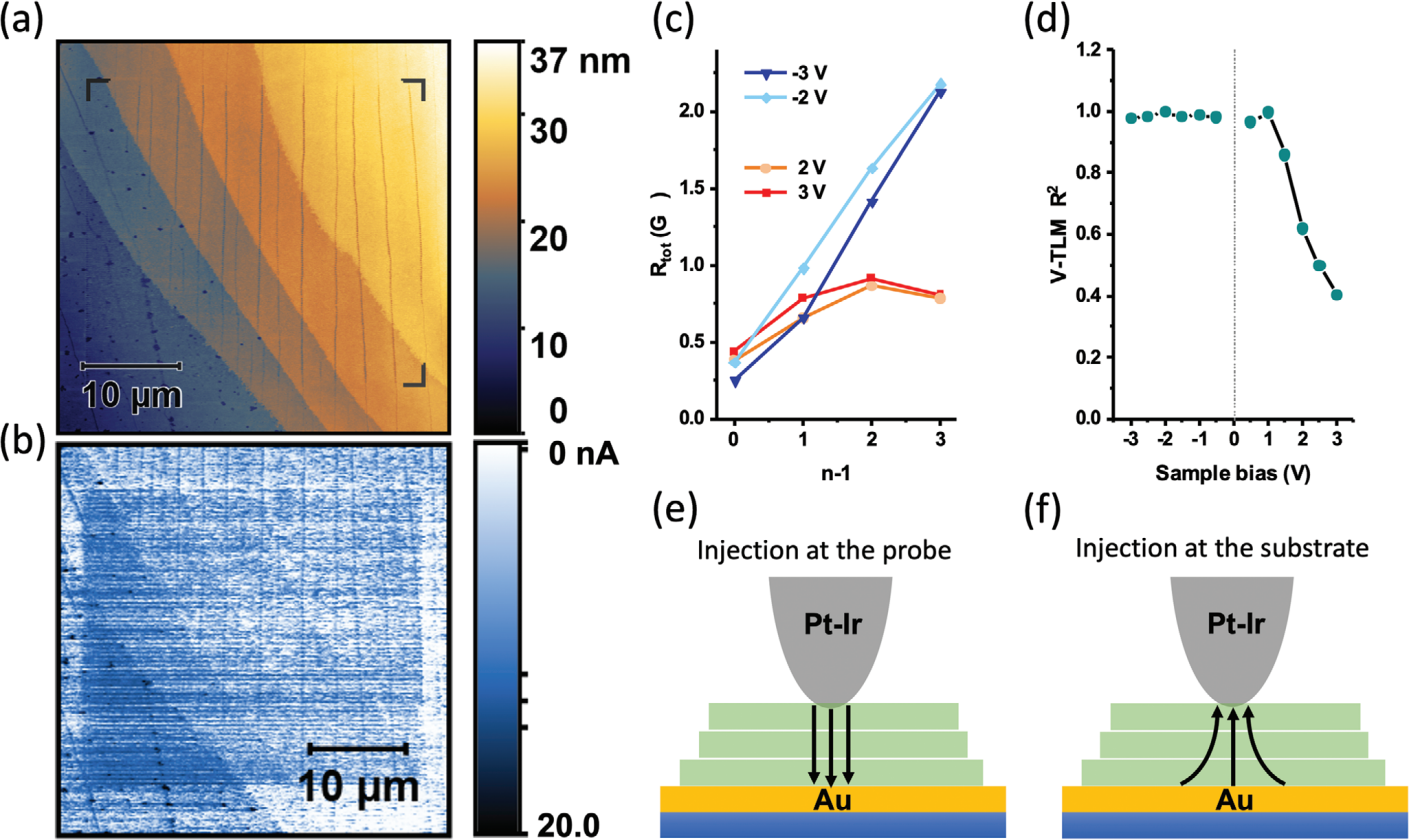
C‐AFM mapping of a) topography and b) current at *V*_sample_ = −1.5 V of a C8‐DNTT‐C8 film, showing irreversible changes to electrical characteristics in the pre‐scanned C8‐DNTT‐C8 film region caused by a high (70 nN) mechanical load applied by the AFM probe. c) V‐TLM plot of the C8‐DNTT‐C8 film at higher positive and negative sample biases and at low mechanical loads of ≈15 nN. d) *R^2^* value of the V‐TLM linear fit of a sample with *n* = 4 at different sample bias values showing the deviation from linearity at higher positive sample bias values. e,f) Proposed current‐injection scenarios for C‐AFM measurements of C8‐DNTT‐C8 single‐crystal thin films at negative sample bias (hole injection from the C‐AFM probe), and positive sample bias (hole injection from the large‐area Au‐coated substrate).

A further increase in mechanical load applied to the sample by the AFM probe (70 nN) caused inelastic deformation of the OSC layer resulting in an irreversible change of the electrical properties of the OSC film. A noticeable increase in the out‐of‐plane current can be observed in the sample area affected by a higher force applied by the probe (**Figure** **6**a,b) which is likely caused by a decreased tunneling distance and/or lower tunneling barrier height due to the disturbed crystal packing of the alkyl chains, thus providing alternative tunneling pathways.

As the in‐plane mobility in C8‐DNTT‐C8 is significantly greater than the out‐of‐plane mobility, the current has the possibility to spread within the molecular plane before propagating to the next layer, thus effectively increasing the electrical contact area and lowering the measured resistance – the effect known as current spreading.^[^ ^27^, ^35^ ^]^ Prominent current spreading may lead to an effective electrical contact area that is several orders of magnitude larger than the mechanical contact area estimated above. Consequently, in case of a pronounced current spreading, a sublinear increase in resistance at higher *n* values would be expected. This, however, was not observed in C‐AFM measurements of crystalline C8‐DNTT‐C8 layers, with the V‐TLM plot showing a linear dependence of *R vs*. (*n –* 1) (Figure 4a).

Furthermore, in a scenario where current spreading played a dominant role, a small increase in the mechanical contact area due to a higher applied mechanical load would cause no significant increase in the measured current, since the effective charge injection and/or collection area at the conductive substrate becomes independent of the probe‐sample force.^[^ ^27^, ^35^ ^]^ However, in our measurements even a small increase in mechanical force applied by the AFM tip induces a large increase in the measured out‐of‐plane current. Additionally, an identical degree of C‐AFM current increase versus applied mechanical load is observed for C8‐DNTT‐C8 layers of different thickness, as shown by the similar slope values in the log‐log plot in Figure 5b. This provides another experimental indication for the absence of significant current spreading effects in our C‐AFM experiments on C8‐DNTT‐C8 at negative sample bias.

Moreover, current spreading relies on the presence of effective lateral conduction pathways within the OSC layer. In previous studies, this has been demonstrated in the C‐AFM study of an OSC monolayer, in which the measured current was higher on the crystalline islands of larger diameter, indicating a very pronounced current spreading.^[^ ^36^ ^]^ However, when the monolayers of the said OSC were mechanically disturbed by applying a high mechanical load with the AFM probe, the out‐of‐plane C‐AFM current in the affected area decreased, indicating that lateral conduction pathways were disrupted and the current spreading was reduced.^[^ ^37^ ^]^ This behavior is contrary to our observations here, where the out‐of‐plane current increased upon the application of a high mechanical load (Figure 6b). The combination of these experimental observations leads to the conclusion that current spreading effects do not play a significant role in our measurements when the C‐AFM probe acts as an injecting electrode (negative sample bias).

At higher positive sample bias, however, the V‐TLM plot significantly deviates from linearity (Figure 6c,d) – a behavior that is indicative of current spreading. This observed behavior might be explained by the fact that under a positive sample bias the hole injection takes place at the large‐area substrate electrode, meaning that the Schottky diode at the Au/OSC interface is reverse‐biased. This leads to an increased resistance at the large‐area electrode which, in turn, promotes hole injection over a larger contact area. Indeed, previous studies have shown that voltage polarity plays a crucial role in C‐AFM measurements, leading to different charge‐propagation pathways and charge‐transport mechanisms.^[^ ^28^ ^]^ We, therefore, propose that conduction here falls into one of two overall scenarios depending on bias polarity: negative bias leading to negligible to no current spreading, and positive bias where current spreading is non‐negligible, as depicted in Figure 6e,f respectively. Current spreading at positive biases hinders the estimation of the interlayer resistivity due to additional uncertainty in the determination of the electrical‐contact and charge‐propagation area. For this reason, only measurements at negative sample bias values, where the V‐TLM plot maintains near‐perfect linearity, were used in further analysis.

By determining the probe/OSC contact area, the interlayer resistivity was calculated to be *ρ*_inter_ = 1.4 ± 0.3 mΩ cm^2^ layer^−1^, which is the main contributor to the bulk resistivity (*ρ*_bulk_) of the OSC when *n* > 1. We define the interlayer resistivity as the electrical resistance per unit area for vertical charge transport between two adjacent layers of the conjugated cores of the semiconductor (which is dominated by the resistance of the bilayer of alkyl side chains attached to the conjugated core). Thus, the bulk resistivity can be estimated by:

(5) $$\rho_{\mathrm{bulk}}\approx \left(n-1\right)\rho_{\mathrm{inter}}$$

Using the C8‐DNTT‐C8 molecular layer thickness of *c* = 3.4 nm, this results in *ρ*_bulk_ = 412 ± 76 kΩ cm. Subsequently, when the thickness of the C8‐DNTT‐C8 is reduced to a single monolayer (*n* = 1), the measured contact resistivity derived from the y‐intercept of the V‐TLM plot reaches $\rho_{C}^{n\;=1}$ = 0.70 ± 0.28 mΩ cm^2^. The total contact resistivity can, therefore, be expressed as:

(6) $$\rho_{C}^{C-\mathrm{AFM}}=\rho_{C}^{n=1}+\left(n-1\right)\rho_{\mathrm{inter}}$$

The charge transport through the Pt‐Ir/C8‐DNTT‐C8/Au junction is predominantly limited by the bulk resistance (*ρ*_bulk_) of the C8‐DNTT‐C8 when the thickness of the OSC is larger than a single monolayer (*n* > 1), and hole injection takes place at the C‐AFM probe electrode. This is evidenced by the y‐intercept values of the V‐TLM plot at negative sample bias being significantly smaller than the values of the V‐TLM slope (Figure 4b,c).

To assess how C‐AFM measurements of *R*_I_ *+ R*_bulk_ compare with contact resistivity measurements in macroscale devices, we fabricated OFET devices with a staggered top contact/bottom gate architecture and different channel lengths. These were based on crystalline C8‐DNTT‐C8 films prepared using a similar process as for the C‐AFM samples. While C‐AFM measurements and OFET devices are not directly comparable, both systems involve metal‐semiconductor interfaces and bulk transport through the OSC. C‐AFM directly probes the out‐of‐plane conductivity by applying a vertical bias across the OSC, whereas OFETs explore in‐plane transport by modulating the charge injection and accumulation through gate voltage control.

**Figure 7:**
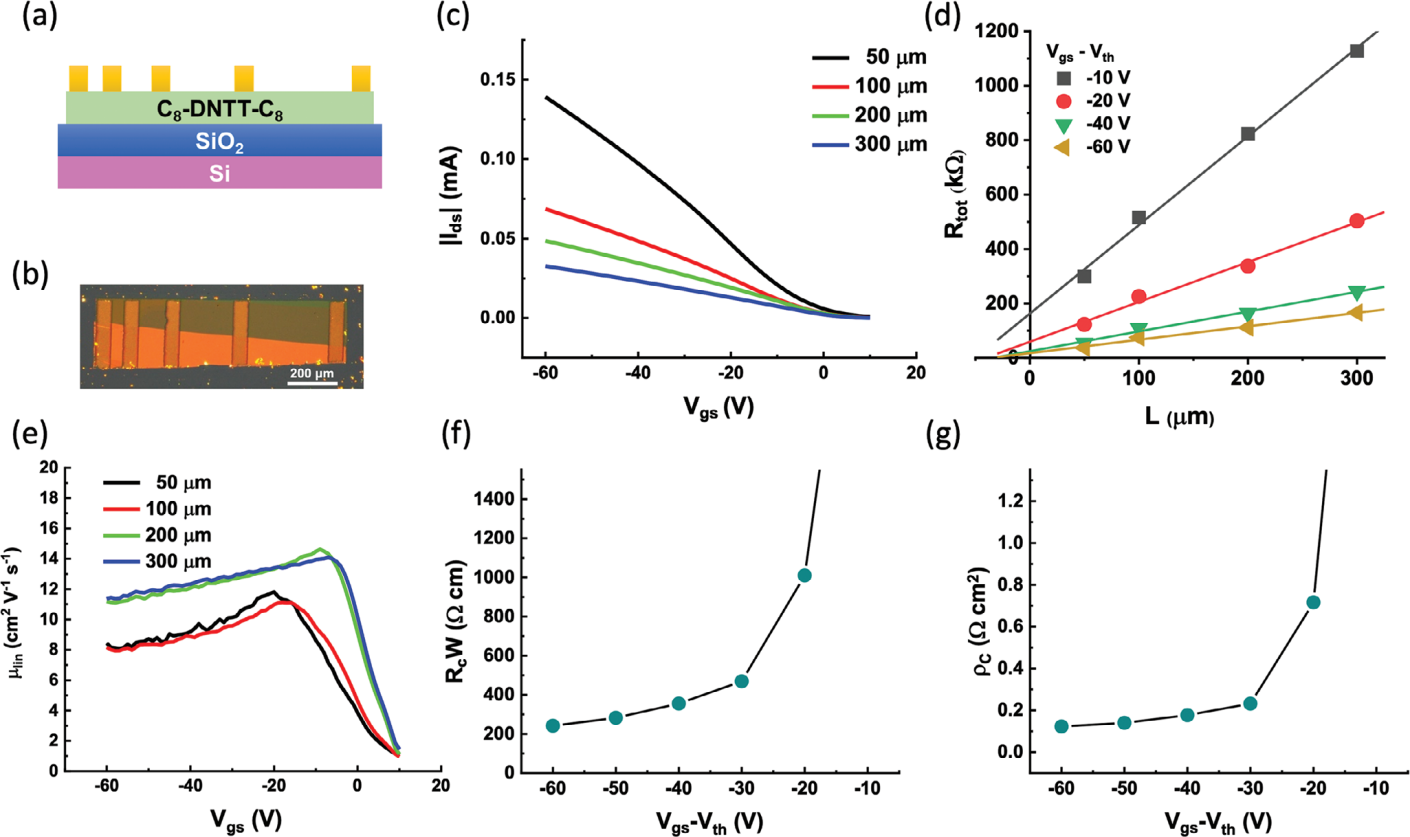
a) Schematics of the OFET devices with different channel lengths for gated transfer length method (TLM) analysis. b) Polarized optical microscopy image of the C8‐DNTT‐C8 OFET devices with *W* = 234 µm and *L* = 50, 100, 200, and 300 µm. c) Linear regime transfer curves (*V*_ds_ = −5 V) of C8‐DNTT‐C8 OFET devices with different channel lengths, employed in this TLM study. d) Total channel resistance *R*_tot_ of the gated C8‐DNTT‐C8 OFET devices as a function of device channel length. e) Gate‐dependent effective charge‐carrier mobility of C8‐DNTT‐C8 OFET devices in the linear regime (*V*_ds_ = −5 V) employed in this study. f) Width‐normalized contact resistance *R*_C_ *W* values extracted from TLM analysis. g) Contact resistivity, $\rho_{C}^{\mathrm{OFET}}$, of the C8‐DNTT‐C8 OFET devices as a function of applied gate bias.

We estimated the contact resistance of the devices via the gated transfer‐length method (TLM). The schematics of the OFET device architecture used in this TLM analysis, and a POM image of the measured devices are shown in **Figure** **7**a,b. The POM image reveals two crystal domains in the OFET device channel, with a single grain boundary aligned parallel to the transport direction and extending through all channels. Due to this alignment, the grain boundary is expected to have minimal impact on charge transport and the extraction of device parameters as a function of channel length. The transfer curves of these devices in the linear regime (*V*_ds_ = −5 V) with different channel lengths are shown in Figure 7c. The devices exhibited low threshold voltages *V*_th_ = −0.2, 2.1, 5.4, and 5.6 V for channel lengths of *L* = 50, 100, 200, and 300 µm, respectively (channel width *W* = 234 µm). The intrinsic charge‐carrier mobility *µ*_0_ = 13.5 cm^2^ V^−1^ s^−1^ was extracted (Figure S4a, Supporting Information), with a maximum effective carrier mobility of *µ*_eff_ = 14.6 cm^2^ V^−1^ s^−1^ recorded in the linear regime (Figure 7e). These values represent some of the highest in‐plane charge carrier mobility of C8‐DNTT‐C8 OFETs reported to date and reflect the high, quasi single‐crystalline nature of our organic films deposited by solution‐shearing.

Figure 7d presents the total source‐drain resistance *R*_tot_ in the linear regime (*V*_ds_ = −5 V) measured at different device channel lengths (*L*). We observed a linear increase in *R*_tot_ with increasing channel length at all gate voltage (*V*_gs_) values. The width‐normalized contact resistance *R*_C_ *W* can then be extracted by extrapolating *L* to zero. Figure 7f shows that the total *R*_C_ *W* decreases with increasing *V*_gs_, reaching a value of *R*_C_ *W* = 241 Ω cm at *V*_gs_ = −60 V. This value is consistent with values reported for similar devices and materials in the literature (Table S1, Supporting Information). Besides the contact resistance and charge‐carrier mobility in the linear regime, the intrinsic charge carrier mobility (µ_0_ = 13.5 cm^2^ V^−1^ s^−1^) and sheet resistance values (*R*_sh_ = 132.2 kΩ sq^−1^ at *V*_gs_ = −60 V) were also determined (Figure S4, Supporting Information). Intrinsic charge carrier moblity of the semiconductor refers to the inherent carrier mobility of the OSC, unaffected by extrinsic factors such as contact resistance or interface imperfections. The measured *R*_C_ *W* value is approximately 2 times higher than the values reported for C10‐DNTT‐C10 monolayer OFET devices with similar architecture and comparable *R*_sh_ and charge‐carrier mobility, when operated under similar gating conditions (*V*_gs_ = −60 V).^[^ ^5^, ^16^ ^]^ This increase is expected, as the channel thickness in our OFET devices measured via AFM is ≈23.3 nm, corresponding to 6–7 molecular layers of C8‐DNTT‐C8. Therefore, the contact resistance in OFET devices presented in this study is expected to be dominated by the bulk resistance of the OSC.

The effective contact area of OFET devices was estimated using the TLM‐based approach, which can be applied when *V*_ds_ ≪ (*V*_gs_ − *V*_th_).^[^ ^5^, ^29^ ^]^ The model describes the carrier injection length *L*_T_ which corresponds to the effective length of the metal contact over which most of the current injection into the channel takes place. The contact resistivity and carrier injection length can be calculated using the following equations^[^ ^5^, ^29^ ^]^:

(7) $$R_{C}W\;\approx R_{\mathrm{bulk}}W=\sqrt{\frac{\rho_{C}}{\mu_{0}C_{i}\left(V_{\mathrm{gs}}-V_{\mathrm{th}}\right)}}$$

(8) $$L_{T}=\sqrt{\rho_{C}\mu_{0}C_{i}\left(V_{\mathrm{gs}}-V_{\mathrm{th}}\right)}\;$$

Figure 7g shows the calculated contact resistivity of C8‐DNTT‐C8 OFET devices, reaching a value of $\rho_{C}^{\mathrm{OFET}}=0.123\;\Omega$ cm^2^ at *V*_gs_ = −60 V with an *L*_T_ value of 10.2 µm, and the effective contact area of *A*_C_ *= W* × *L*_T_ = 2392 µm^2^ (Figure S3, Supporting Information). Notably, the value of $\rho_{C}^{\mathrm{OFET}}$ includes both the resistances of the interface and the bulk, which cannot be distinguished using the conventional gated transfer length method.

The expected contact resistivity value derived from the V‐TLM analysis based on C‐AFM measurements for the C8‐DNTT‐C8 OFET device with $n=6.5\;\;\mathrm{is}\;\;\rho_{C}^{C-\mathrm{AFM}}=8.4\pm 1.4\;m\Omega \;\;{\mathrm{cm}}^{2}$, which is an order of magnitude lower than the $\rho_{C}^{\mathrm{OFET}}$ value obtained through macroscale OFET device measurements. The discrepancy between the *ρ*_C_ values in macroscale OFET devices and the C‐AFM‐based V‐TLM approach may originate from the significantly different nature of the two devices. While electrical contact in C‐AFM is established via a gentle mechanical approach, the Au contacts in OFET devices were deposited via thermal evaporation, which has been shown to cause damage to the OSC film, leading to higher contact resistance.^[^ ^38^, ^39^ ^]^

Furthermore, the conductive coating of the AFM probes used in our C‐AFM experiments was based on a Pt‐based alloy. It has been reported that using mechanically transferred Pt contacts may reduce the contact resistivity in OFET devices based on C10‐DNTT‐C10 by over 3 times compared to the devices with mechanically transferred Au contacts, and over 40 times compared to the Au electrodes deposited by thermal evaporation. This reduction in contact resistivity arises from a Pt‐catalyzed interaction between the metal and the OSC via orbital hybridization.^[^ ^39^ ^]^ In fact, in their work on OFET devices based on monolayer C10‐DNTT‐C10 with mechanically applied Pt electrodes, Zeng, J. et al. reported contact resistivity of ρ_C_ =  1.26 mΩ cm^2^, which is only 1.8 times higher than the $\rho_{C}^{C-\mathrm{AFM}}=0.70\;\pm \;0.28\;m\Omega \;{\mathrm{cm}}^{2}$ determined by the V‐TLM approach in the C‐AFM measurements of C8‐DNTT‐C8 at *n* = 1 here.^[^ ^39^ ^]^ Part of this difference can be accounted for by the increased resistivity due to the longer insulating alkyl chains in C10‐DNTT‐C10. This showcases that our C‐AFM‐based V‐TLM approach does indeed enable accurate estimation of contact resistance parameters and material properties.

Due to the discussed enhanced probe/OSC interactions and the damage‐free mechanical application of electrical contact, we consider the $\rho_{C}^{C-\mathrm{AFM}}$ values obtained via the herein‐described C‐AFM measurements to represent the lower boundary of achievable contact resistivity in staggered architecture devices. Taking these factors into account, we consider the $\rho_{C}^{C-\mathrm{AFM}}$ values determined via V‐TLM to fall within the range of contact resistivity values determined using other experimental techniques, thus supporting the validity of our C‐AFM‐based V‐TLM technique. Furthermore, the power of this approach lies in its capability to experimentally measure the interlayer resistivity of an OSC, without being marred by interfacial contact effects. This method enables one to separately study the contributions to the total contact resistivity arising from the OSC/metal interface and the bulk resistivity of the OSC. This opens the pathway to gaining insights into the interplay between the molecular structure and the out‐of‐plane resistivity of OSC materials.

## 3 Conclusion

In this study, we used a solution shearing method to fabricate large‐area, single‐crystal thin films of C8‐DNTT‐C8, exhibiting a terraced topography on Au‐coated Si/SiO_2_ substrates. We examined these films by employing conductive atomic force microscopy (C‐AFM) to measure local changes in out‐of‐plane electrical characteristics as a function of number of molecular layers *n* in the film. This precise control over the current‐collection data, relative to current propagation distance, enabled us to investigate the charge transport properties of these multi‐layered, single‐crystal films in the out‐of‐plane direction.

We observed a linear increase in resistance as a function of the number of molecular layers and modeled this electrical behavior using an equivalent circuit, consisting of multiple tunneling barriers connected in series. In this context, we have developed a vertical transfer length method (V‐TLM) that encompasses both the experimental approach and the proposed model to estimate the out‐of‐plane resistivity of a single molecular layer, using C‐AFM current‐mapping data.

The comparison of the interface and interlayer resistivity values, derived from V‐TLM, demonstrated a relatively close match with those obtained from TLM analysis in OFET devices with different channel lengths. C‐AFM is shown to be a powerful tool for investigating the out‐of‐plane charge transport in molecular OSC thin films in a sample geometry easily comparable to that of an operating staggered‐architecture OFET, hence providing a versatile method for extracting relevant parameters related to contact resistivity and the out‐of‐plane charge transport.

Investigations of small‐molecule OSC thin films via C‐AFM provide a powerful tool for advancing the development of high‐performance electronic devices featuring staggered architectures, wherein vertical (out‐of‐plane) charge transport is pivotal for device functionality. Our method developed here has the potential to accelerate the screening of promising OSC compounds, thus bypassing the need to fabricate complex devices to assess the electrical properties of these materials. Because of the gentle and controlled surface contact that is established in C‐AFM, the technique provides quantitative values of the minimum contact resistivities that could be expected in optimized FET devices, when care is taken to minimize degradation of the interface upon metal contact deposition on the OSC.

## 4 Experimental Section

### Sample Fabrication

C8‐DNTT‐C8 was synthesized according to a literature procedure.^[^ ^21^ ^]^ 1,2‐dichlorobenzene (DCB) (assay >99.8%, water <0.0020%, residue <0.0005%) was purchased from Romil. Highly doped Si wafers with 300‐nm thermally grown SiO_2_ coated with a uniform, thermally evaporated Cr/Au (5/25 nm) film were used as substrates for C‐AFM sample fabrication. The films of C8‐DNTT‐C8 were fabricated by a meniscus‐guided solution shearing method from a 1.0 g L^−1^ solution of C8‐DNTT‐C8 in DCB at a shearing rate of 5 µm s^−1^ with the substrates held at 65 °C.

OFET devices with different channel lengths were prepared in a bottom‐gate/top‐contact configuration on Si wafer substrates with a 300 nm thermally grown SiO_2_ layer (areal capacitance *C*_i_ = 10.6 nF cm^−2^). These substrates were washed, using an ultrasonic bath, in diluted Decon 90 detergent solution, deionized water, acetone, and 2‐propanol for 10 min each, followed by an oxygen plasma treatment at 300 W for 10 min. The films of C8‐DNTT‐C8 were deposited by solution shearing from a 1.0 g L^−1^ solution of C8‐DNTT‐C8 in DCB, with the substrates held at 70 °C and at a shearing rate of 8 µm s^−1^. Top‐contact gold electrodes (30 nm) were evaporated through a shadow mask at 2 × 10^−6^ mbar and a 0.15 Å s^−1^ deposition rate.

Transistor devices were characterized using an Agilent 4155B Semiconductor Parameter Analyzer at room temperature. Sample fabrication and device measurements were carried out in a N_2_‐filled glovebox (O_2_ and H_2_O < 5 ppm). Polarized optical microscopy measurements were performed with a Nikon ECLIPSE LV100N POL microscope to characterize the crystalline C8‐DNTT‐C8 films.

### Atomic Force Microscopy Measurements

AFM measurements were performed in contact mode using an Asylum Research MFP‐3D AFM system equipped with an ORCA transimpedance amplifier (Oxford Instruments). Pt‐Ir‐coated conducting probes (SCM‐PIT‐V2 from Bruker) possessing a nominal tip radius of 25 nm, a nominal spring constant of 3 N m^−1^, and a resonant frequency of 75 kHz were used for C‐AFM measurements.

During C‐AFM measurements the conducting probe was grounded, and bias was applied to the sample. The deflection sensitivity was experimentally calibrated by measuring force curves on a Si/SiO_2_ wafer sample. The spring constant was then calibrated by thermal tuning in air. The Young's modulus of C8‐DNTT‐C8 was calculated using the Hertz model.^[^ ^40^ ^]^ All AFM measurements were performed under ambient conditions.

### Data Analysis

The processing and analysis of experimental C‐AFM data have been performed using Gwyddion and Python.^[^ ^41^ ^]^ AFM topography images have been processed using the median of differences row alignment method and leveled by fitting a three‐point plane. The height data was plotted as a histogram with 500 bins per dataset. The height histogram data was fitted by a multimodal normal distribution to extract the mean (central height (*Z*_C_) of a specific crystal layer) and standard deviation (*σ*) values of each crystal layer's height, which are marked by the error bars in Figure 2d. In the *I vs. Z* plot, the points and error bars depict the mean and standard deviation values respectively of electrical current in C‐AFM measurements for every individual crystal layer in the C‐AFM scan. TLM and *R vs. n* data were fitted with a standard linear least‐squares regression using OriginPro 2021.

CCDC 1845516 contains the supplementary crystallographic data for this paper. These data can be obtained free of charge from The Cambridge Crystallographic Data Centre via www.ccdc.cam.ac.uk/data_request/cif.

## Conflict of Interest

H.S. is Chief Scientist of FlexEnable Ltd.

## Supporting information



Supporting Information

## Data Availability

The data that support the findings of this study are available from the corresponding author upon reasonable request.
