# Supplementary material for: Probing Out‐Of‐Plane Charge Transport in Organic Semiconductors Using Conductive Atomic Force Microscopy
Source: Adv Mater. 2024 Dec 26;37(7):2418694. doi: 10.1002/adma.202418694 (PMC11837897; doi:10.1002/adma.202418694)
Supplement: Supplementary file 1 — Supporting Information [file ADMA-37-2418694-s001.docx]

Supporting Information

Probing Out-of-Plane Charge Transport in Organic Semiconductors Using Conductive Atomic Force Microscopy

Mindaugas Gicevičius^1^*, Haoxin Gong^1^, Nicholas Turetta^2^, William Wood^1^, Martina Volpi^3^, Yves Geerts^3,4^, Paolo Samorì^2^, Henning Sirringhaus^1^*

M. Gicevičius, H. Gong, W. Wood, H. Sirringhaus

Cavendish Laboratory, University of Cambridge, JJ Thomson Avenue, CB3 0HE Cambridge, United Kingdom
E-mail: mg980@cam.ac.uk, hs220@cam.ac.uk

N. Turetta, P. Samorì
Université de Strasbourg, CNRS, ISIS UMR 7006, 8 allée Gaspard Monge, F-67000 Strasbourg, France

M. Volpi, Y. Geerts

Laboratoire de Chimie des Polymères, Université Libre de Bruxelles (ULB), Boulevard du Triomphe, CP 206/01, 1050 Bruxelles, Belgium

Y. Geerts

International Solvay Institutes of Physics and Chemistry, CP 206/01, Université Libre de Bruxelles (ULB), Boulevard du Triomphe, CP231, 1050 Bruxelles, Belgium

Supporting Information Table of Contents

AFM surface morphology of thermally evaporated Cr/Au layers 3

AFM force-distance curve of C8-DNTT-C8 4

C-AFM measurements of thicker C8-DNTT-C8 films 5

Extraction of contact resistance from TLM measurements on OFET devices 6

Intrinsic charge-carrier mobility and sheet resistance of OFET devices 8

Carrier injection length in OFET devices 9

References 9

# **AFM surface morphology of thermally evaporated Cr/Au layers**

The surface morphology of Cr/Au layers on Si/SiO_2_ substrates were investigated using atomic force microscopy. **Figure S1** shows a 10 µm × 10 µm AFM topography scan area of Cr/Au film. Some surface contamination defects of unknown origin with a height up to 18 nm are present, which are the likely cause of pinholes in the first few molecular layers of C8-DNTT-C8 films prepared by solution shearing, as observed in C-AFM topography and current scans in the main text. The RMS roughness value of 796 pm was calculated in the 10 µm × 10 µm scan area including the surface defects, with the RMS roughness value reaching 550 pm when the sharp defects were excluded. This indicates the low roughness of thermally evaporated Cr/Au layers which is favorable for the self-assembly of C8-DNTT-C8 molecules.

**Figure S1.** (a) AFM topography scan of thermally evaporated Cr/Au (5/25 nm) layers on Si/SiO_2_ wafer. (b) AFM height profile of Cr/Au film including the surface defects.

# **AFM force-distance curve of C8-DNTT-C8**

Elastic modulus of single-crystal C8-DNTT-C8 films was extracted from the force-distance curves (n = 16) recorded using atomic force microscope (**Figure S2**). The elastic modulus values were extracted using the Hertz model of elastic contacts.^[1]^


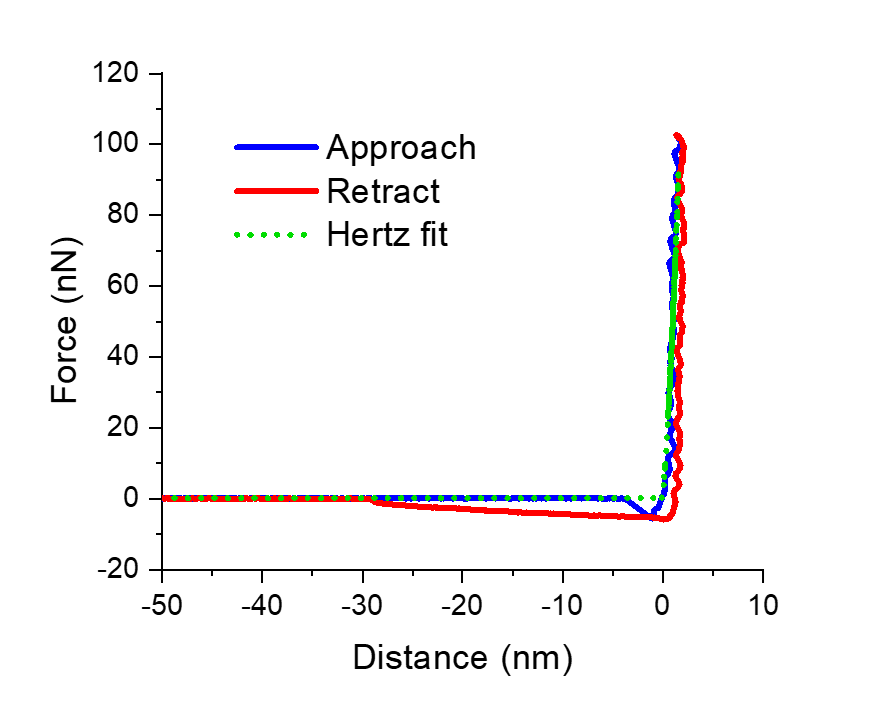


**Figure S2.** AFM force-distance curve of single-crystal C8-DNTT-C8 film prepared by the solution shearing method.

# **C-AFM measurements of thicker C8-DNTT-C8 films**

C8-DNTT-C8 films with larger thickness values (*n* > 4) were investigated using C-AFM by increasing mechanical load applied by the probe (*P* ≈ 29 nN), thus effectively increasing the electrical contact area. **Figure S3** shows two separate C-AFM topography (Figure S3 a, d) and current (Figure S3 b, e) mapping measurements of C8-DNTT-C8 films with *n* = 6 (Sample A), and *n* = 7 (Sample B). Figure S3 panels c and f show *I vs. Z* plots for C-AFM measurements of Sample A and Sample B, respectively.

Figure S3. C-AFM surface topography and electrical current maps as well as *I vs. Z* plots of single crystal C8-DNTT-C8 thin films with higher thicknesses, sample A (*n* = 6): panels (a-c), sample B (*n* = 7) panels (d-f), respectively.

# **Extraction of contact resistance from TLM measurements on OFET devices**

# The extracted value of *R*_C_*W* = 241 Ω cm from the device shown in Fig. 7 is consistent with contact resistance values reported for similar devices and materials in the literature. Table S1 provide a comparison of our value with some literature reports.

**Table S1.** Comparison of contact resistance values with previously reported OFET devices based on single-crystal OSC films.

| **Material** | **Layer thickness (nm)** | **Contacts** | **Dielectric** | **R_C_W (Ω cm)** | **Ref** |
| --- | --- | --- | --- | --- | --- |
| C8-DNTT-C8 | 23.5  6-7 layers | Au (evaporated) | SiO_2,_ 300 nm  C_i_ = 10.6 nF cm^-2^ | 241  (V_gs_-V_th_= -60 V) | This work |
| C10-DNTT-C10 | monolayer | Au (laminated) | SiO_2_, 300 nm  C_i_ = 11 nF cm^-2^ | 75  (V_gs_-V_th_= -80V) | (1) |
|  | 2 layers |  |  | 186  (V_gs_-V_th_= -80 V) |  |
| C10-DNTT-C10 | monolayer | Au (laminated) | SiO_2,_ 300 nm  C_i_ = 10.6 nF cm^-2^ | 89.9  (V_gs_-V_th_= -80 V) | (2) |
|  |  | Au (laminated) | HfO_2_  C_i_ = 450 nF cm^-2^ | 105  (V_gs_-V_th_= -3.0V) |  |
| C10-DNTT-C10 | monolayer | Au (evaporated) | HfO_2_  C_i_ = 510 nF cm^-2^ | 857.5  (V_gs_-V_th_= -3.0V) | (3) |
|  |  | Au (laminated) |  | 60.4 |  |
|  |  | Pt (laminated) |  | 14.0 |  |
| Ph-BTBT-C10 | n/a | Au (laminated) |  | 206.6 |  |
|  |  | Pt (laminated) |  | 139.2 |  |
| C8-BTT-C8 | n/a | Au (laminated) |  | 95.2 |  |
|  |  | Pt (laminated) |  | 67.0 |  |
| C9-DNBDT-NW | monolayer | Au (laminated) | SiO_2,_ 100 nm  C_i_ = 34.5 nF cm^-2^ | 175 (V_gs_-V_th_= -20 V) | (4) |
| C9-DNBDT-NW | 2 layers | Au (evaporated),  F4TCNQ doped | SiO_2,_ 100 nm  C_i_ = 34.5 nF cm^-2^ | 46.9 (V_gs_-V_th_= -30 V) | (5) |
|  | 3 layers |  |  | 260  (V_gs_-V_th_= -30 V) |  |
| BEPA | 20 | Au (laminated) | SiO_2_, 300 nm  C_i_ = 10.6 nF cm^-2^ | 335  (V_gs_-V_th_= -40 V) | (6) |

**Table references:**

1. B. Peng, K. Cao, A. H. Y. Lau, M. Chen, Y. Lu, P. K. L. Chan, Crystallized Monolayer Semiconductor for Ohmic Contact Resistance, High Intrinsic Gain, and High Current Density. *Adv. Mater.* **32**, 2002281 (2020).

2. M. Chen, B. Peng, R. A. Sporea, V. Podzorov, P. K. L. Chan, The Origin of Low Contact Resistance in Monolayer Organic Field‐Effect Transistors with van der Waals Electrodes. *Small Sci.* **2**, 2100115 (2022).

3. J. Zeng, D. He, J. Qiao, Y. Li, L. Sun, W. Li, J. Xie, S. Gao, L. Pan, P. Wang, Y. Xu, Y. Li, H. Qiu, Y. Shi, J.-B. Xu, W. Ji, X. Wang, Ultralow contact resistance in organic transistors via orbital hybridization. *Nat. Commun.* **14**, 324 (2023).

4. T. Makita, A. Yamamura, J. Tsurumi, S. Kumagai, T. Kurosawa, T. Okamoto, M. Sasaki, S. Watanabe, J. Takeya, Damage-free Metal Electrode Transfer to Monolayer Organic Single Crystalline Thin Films. *Sci. Rep.* **10**, 4702 (2020).

5. A. Yamamura, S. Watanabe, M. Uno, M. Mitani, C. Mitsui, J. Tsurumi, N. Isahaya, Y. Kanaoka, T. Okamoto, J. Takeya, Wafer-scale, layer-controlled organic single crystals for high-speed circuit operation. *Sci. Adv.* **4**, eaao5758 (2018).

6. Y. Sun, X. Shi, Y. Yu, Z. Zhang, M. Wu, L. Rao, Y. Dong, J. Zhang, Y. Zou, S. You, J. Liu, M. Lei, C. Liu, L. Jiang, Low Contact Resistance Organic Single‐Crystal Transistors with Band‐Like Transport Based on 2,6‐Bis‐Phenylethynyl‐Anthracene. *Adv. Sci.* **11**, 2400112 (2024).

# **Intrinsic charge-carrier mobility and sheet resistance of OFET devices**

We also extracted the intrinsic charge carrier mobility and sheet resistance from the TLM measurements on OFET devices. The intrinsic hole mobility in the linear regime presented in **Figure S4a** was calculated from gate bias dependent sheet resistance (Figure S4b) values extracted from TLM measurements in OFET devices with different channel lengths using the Equation (S1):^[2–4]^

|  | $R_{\mathrm{sh}}\approx\frac{1}{\mu_{0}C_{i}(V_{\mathrm{gs}}-V_{\mathrm{th}})}$ | (S1) |
| --- | --- | --- |


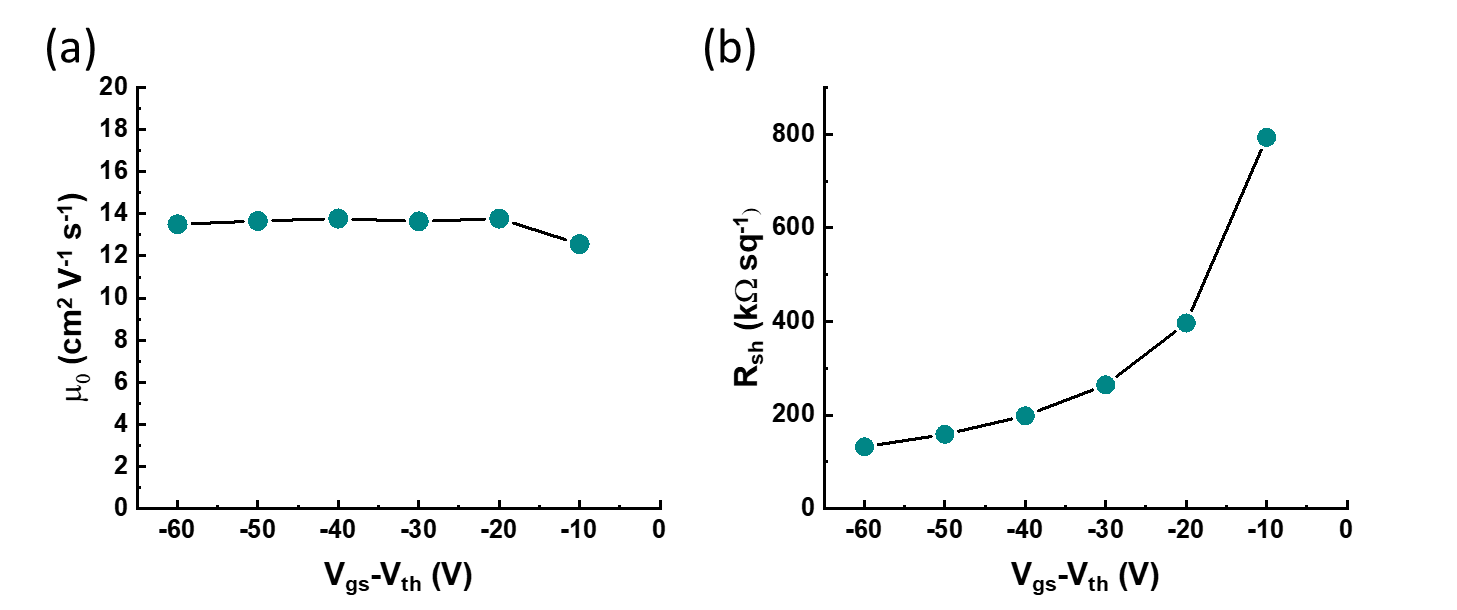


Figure S4. Intrinsic hole mobility in the linear regime (a) and sheet resistance (b) of OFET devices with different channel lengths based on crystalline thin films of C8-DNTT-C8 as a function of applied gate voltage.

# **Carrier injection length in OFET devices**


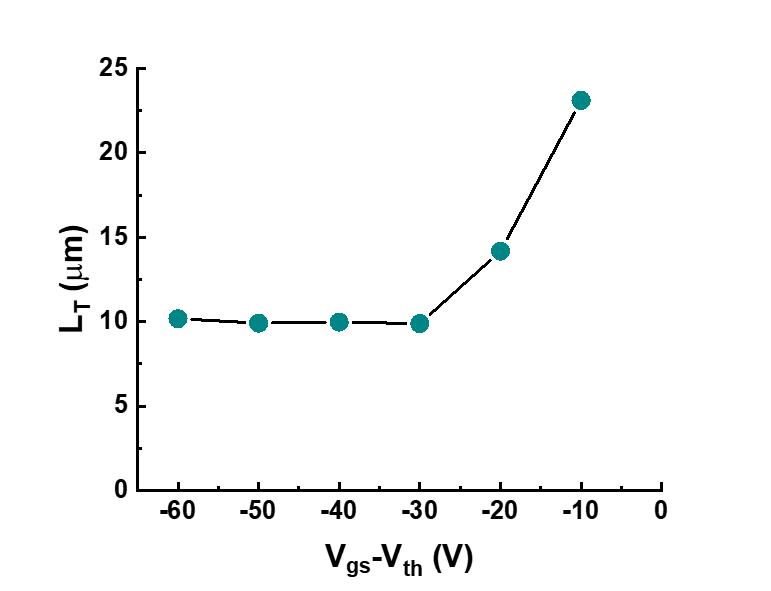


Figure S5. Carrier injection length *L*_T_ in C8-DNTT-C8 OFET devices derived from the TLM analysis as a function of gate bias.

# **SI References**

[1] J. B. Pethica, W. C. Oliver, *Phys. Scr.* **1987**, *T19A*, 61.

[2] K. D. Jung, Y. C. Kim, B. G. Park, H. Shin, J. D. Lee, *IEEE Trans. Electron Devices* **2009**, *56*, 431.

[3] M. Chen, B. Peng, R. A. Sporea, V. Podzorov, P. K. L. Chan, *Small Sci.* **2022**, *2*, 2100115.

[4] M. Waldrip, O. D. Jurchescu, D. J. Gundlach, E. G. Bittle, *Adv. Funct. Mater.* **2020**, *30*, 1904576.
